# Previously implanted mitral surgical prosthesis in patients undergoing transcatheter aortic valve implantation: Procedural outcome and morphologic assessment using multidetector computed tomography

**DOI:** 10.1371/journal.pone.0226512

**Published:** 2019-12-26

**Authors:** Makoto Tanaka, Ryo Yanagisawa, Fumiaki Yashima, Takahide Arai, Masahiro Jinzaki, Hideyuki Shimizu, Keiichi Fukuda, Yusuke Watanabe, Toru Naganuma, Shinichi Shirai, Motoharu Araki, Norio Tada, Futoshi Yamanaka, Akihiro Higashimori, Kensuke Takagi, Hiroshi Ueno, Minoru Tabata, Kazuki Mizutani, Masanori Yamamoto, Kentaro Hayashida, on behalf of the OCEAN-TAVI investigators

**Affiliations:** 1 Department of Cardiology, Keio University School of Medicine, Tokyo, Japan; 2 Department of Radiology, Keio University School of Medicine, Tokyo, Japan; 3 Department of Cardiovascular Surgery, Keio University School of Medicine, Tokyo, Japan; 4 Department of Cardiology, Teikyo University School of Medicine, Tokyo, Japan; 5 Department of Cardiology, New Tokyo Hospital, Matsudo, Japan; 6 Department of Cardiology, Kokura Memorial Hospital, Kitakyushu, Japan; 7 Department of Cardiology, Saiseikai Yokohama City Eastern Hospital, Yokohama, Japan; 8 Department of Cardiology, Sendai Kousei Hospital, Sendai, Japan; 9 Department of Cardiology, Shonan Kamakura General Hospital, Kamakura, Japan; 10 Department of Cardiology, Kishiwada Tokushukai Hospital, Kishiwada, Japan; 11 Department of Cardiology, Ogaki Municipal Hospital, Ogaki, Japan; 12 Second Department of Internal Medicine, University of Toyama, Toyama, Japan; 13 Department of Cardiovascular Surgery, Tokyo Bay Urayasu Ichikawa Medical Center, Urayasu, Japan; 14 Department of Cardiovascular Medicine, Osaka City University Graduate School of Medicine, Osaka, Japan; 15 Department of Cardiology, Toyohashi Heart Center, Toyohashi, Japan; Thomas Jefferson University, UNITED STATES

## Abstract

Transcatheter aortic valve implantation (TAVI) in the presence of a preexisting mitral prosthesis is challenging and its influence on the morphology of mitral prosthesis and the positioning of transcatheter heart valve (THV) is unknown. We assessed the feasibility of TAVI for patients with preexisting mitral prostheses, its influence on mitral prosthesis morphology, and the positional interaction between a newly implanted THV and mitral prosthesis using serial multidetector computed tomography (MDCT). Thirty-one patients with preexisting mitral prosthesis undergoing TAVI were included. MDCT was performed before and after TAVI. Thirty patients successfully underwent TAVI without interference from preexisting mitral prosthesis. Although opening disturbance of the mechanical mitral prosthesis by the THV edge was observed in 1 patient, the patient was managed conservatively. No THV embolization occurred. THV shift during deployment occurred in 9 patients and was predicted by a larger aortic annulus area (odds ratio: 1.24 per 10 mm^2^, 1.03–1.49, *p* = 0.02), possibly because of large THVs. The mitral mean pressure gradient was slightly higher after TAVI (3.7 vs. 4.3 mmHg, *p* = 0.002), whereas the mitral regurgitation grade was similar. MDCT showed that the size of the mitral prosthesis housing was unchanged after TAVI. The median distance between the mitral prosthesis and THV was 2.6 mm. The postprocedural angle between the mitral prosthesis and THV was larger than the preprocedural angle between the mitral prosthesis and the left ventricular outflow tract (64° vs. 61°, *p* = 0.03). Thus, TAVI is feasible in the case of preexisting mitral prosthesis. Serial MDCT demonstrated favorable THV positioning and unchanged mitral prosthesis morphology after TAVI.

## Introduction

Transcatheter aortic valve implantation (TAVI) is an option for patients with severe aortic stenosis with a certain risk for complications of surgical aortic valve replacement [1–3]. It may also benefit patients who have undergone previous cardiac surgery (e.g., coronary bypass, mitral valve replacement [MVR]) [4].

For patients who have undergone MVR and have a previously implanted mitral prosthesis, TAVI is considered technically challenging because the rigid housing or protruding stem of the prosthesis might interfere with the optimal positioning of the transcatheter heart valve (THV) and increase the risk of valve embolization [4–6]. Furthermore, the newly implanted THV could interfere with the preexisting mitral prosthesis [7,8].

One registry and several case series have reported the feasibility of TAVI in this situation [4–6,9–16]. A previous report demonstrated favorable outcomes in 91 patients with preexisting mitral prostheses who underwent TAVI [6]. It also reported a relatively high occurrence of THV embolization (6.7%) and suggested that a short distance between the aortic annulus and mitral prosthesis was a potential risk factor for THV embolization. It has commented that new-generation TAVI devices are considered likely to reduce the risk of THV embolization. Another concern was the increasing mitral pressure gradient after TAVI, which was reported in the same study [6]. The mechanism behind this phenomenon, including whether the function or morphology of mitral prosthesis is influenced by TAVI, is unclear.

We assessed the feasibility of TAVI in patients with previously implanted mitral prostheses. We also assessed anatomical features in relation to the specific procedural risk, the influence of TAVI on the morphology of the prosthesis, and the positional relationship between newly implanted THV and the prosthesis using preprocedural and postprocedural multidetector computed tomography (MDCT).

## Materials and methods

### Study population and procedure

We analyzed data from the Optimized transCathEter vAlvular iNtervention—Transcatheter Aortic Valve Implantation (OCEAN-TAVI) registry, an ongoing multicenter, prospective registry of patients undergoing TAVI for severe aortic stenosis in 14 Japanese institutions [17,18]. The OCEAN-TAVI registry protocol is registered in the University Hospital Medical Information Network (UMIN000020423). Patients with non-calcified aortic valves and failed aortic bioprostheses were excluded. The protocol was initially approved by the institutional review board committee of the Keio University School of Medicine and was subsequently approved by the ethical committee of each center. All included patients provided written informed consent before the procedure.

Between October 2013 and November 2017, consecutive patients undergoing TAVI were prospectively registered. Patients who had undergone surgical MVR before a TAVI procedure with any type of mechanical valve or bioprosthetic valve were included. Patients who had undergone mitral valve plasty or annular plasty without the use of any valve prosthesis were excluded.

Patients were implanted with balloon-expandable SAPIEN XT or 3 (Edwards Lifesciences, Inc., Irvine, CA, USA) or self-expandable CoreValve Evolut R (Medtronic, Inc., Minneapolis, MN, USA). THV sizes of 20, 23, 26, or 29 mm for SAPIEN XT/3, and 23, 26, or 29 mm for Evolut R were chosen. The transfemoral or transapical approach site was used for SAPIEN XT, and the transfemoral approach site was used for SAPIEN 3 and Evolut R. MDCT and transthoracic echocardiography (TTE) were used to choose appropriate THV and to determine the approach.

### MDCT and TTE measurement for the mitral prosthesis

**Fig 1:**
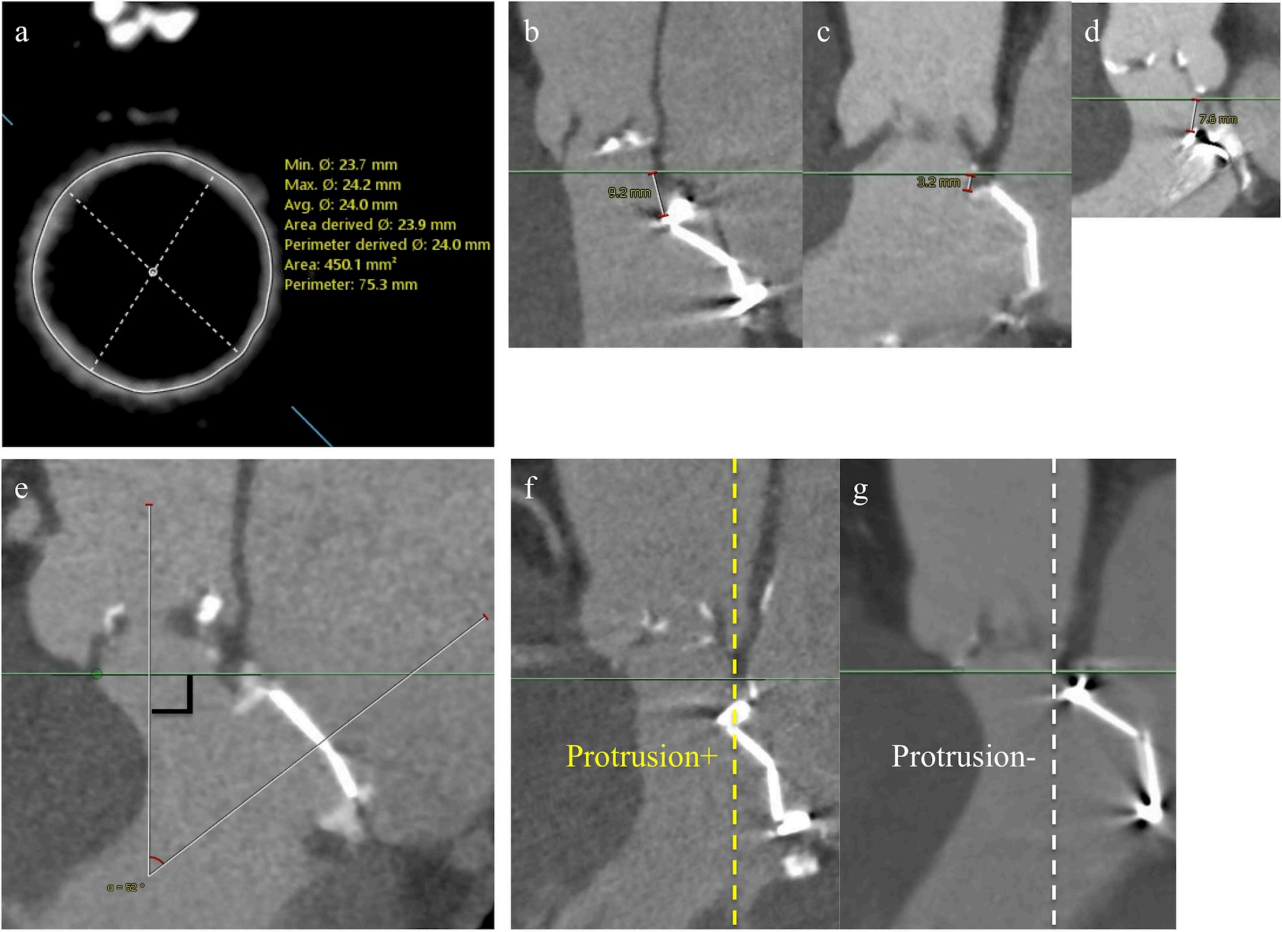
Preprocedural MDCT measurement. (a) Size of the mitral prosthesis housing. (b and c) The distance between the aortic annulus and mitral prosthesis housing. (d) The distance between the aortic annulus and the stem of the mitral bioprosthesis. (e) The angle between the mitral prosthesis and LVOT. (f and g) Assessment of mitral prosthesis protrusion to the LVOT. LVOT = left ventricular outflow tract; MDCT = multidetector computed tomography.

In addition to conventional MDCT measurement of the aortic valve complex, we measured the 1) size of the mitral prosthesis housing (Fig 1A). We measured the area, maximal diameter, and minimal diameter via the mitral prosthesis axial view; 2) distance between the aortic annulus (= virtual basal ring [19]) and the mitral prosthesis housing at the nearest point, via the sagittal view of the heart, to assess the positional relationship between them (Fig 1B and 1C). For bioprosthetic mitral valves, we also measured the distance between the aortic annulus and stem of the mitral bioprosthesis protruding to the left ventricular outflow tract (LVOT) (Fig 1D); 3) angle between the mitral prosthesis and LVOT (Fig 1E) that corresponded to the line perpendicular to the aortic annulus, via the sagittal view of the heart that crosses the center of the mitral prosthesis; and 4) whether the mitral prosthesis housing protruded to the LVOT via the sagittal view that visualized the part of mitral prosthesis closest to LVOT. We defined LVOT housing protrusion as any part of the housing that exceeded the perpendicular line down from the mitral side of the aortic annulus (Fig 1F). Otherwise, it was defined as no protrusion (Fig 1G).

**Fig 2:**
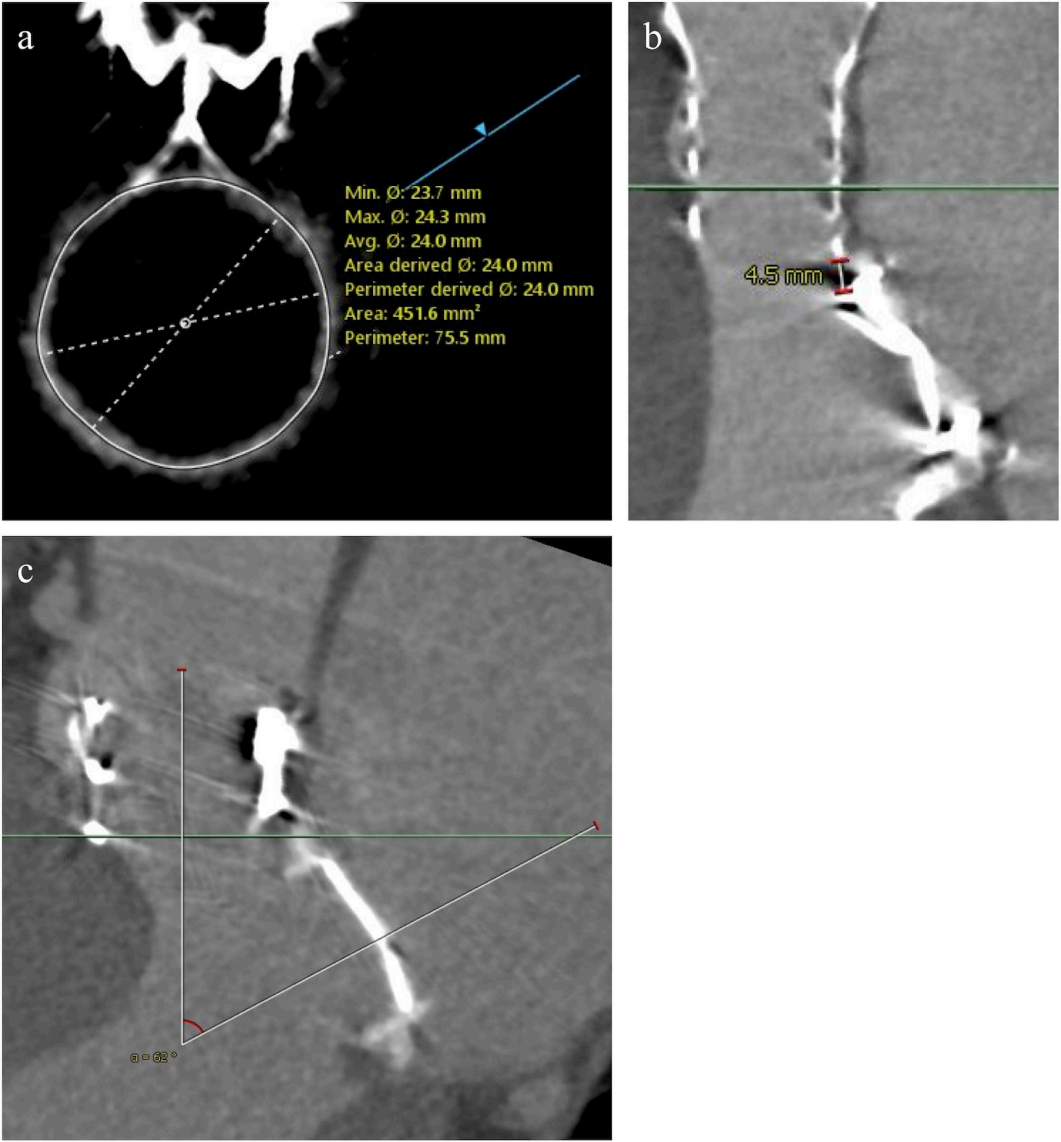
Postprocedural MDCT measurement. (a) Size of the mitral prosthesis housing. (b) The distance between the newly implanted THV and mitral prosthesis housing. (c) The angle between the mitral prosthesis and newly implanted THV. LVOT = left ventricular outflow tract; MDCT = multidetector computed tomography; THV = transcatheter heart valve.

We also performed MDCT within 1 month after the procedure. Non-contrast MDCT was performed for patients with renal insufficiency or allergy to contrast media. Moreover, MDCT was not performed for patients with hemodynamic instability and other factors that precluded its use. We measured: 1) size of the mitral prosthesis (Fig 2A); 2) distance between the newly implanted THV and the mitral prosthesis housing at the nearest point (Fig 2B) to assess if the THV was implanted at the proper position; and 3) the angle between the mitral prosthesis and the newly implanted THV **(**Fig 2C), via the sagittal view of the heart that crosses the mitral prosthesis center.

All MDCT measurements were performed during the systolic phase, which corresponded to 20%–40% of the R-R interval. Representative MDCT images of bioprosthetic valves are provided in S1 Fig.

TTE was performed pre- and postprocedurally. Where possible, TTE data were also recorded 6 months after the procedure. We applied the mean pressure gradient based on the continuous wave Doppler and regurgitation grade of the color Doppler for functional assessment of mitral prosthesis.

### Study endpoints

We assessed procedural outcomes and in-hospital complications, including procedural success, THV interference with mitral prosthesis, valve embolization, THV-in-THV deployment, THV shift during deployment, THV function assessed by TTE, disabling stroke, bleeding complication, new or worsened cardiac conduction disturbance, new pacemaker implantation, acute kidney injury, vascular complication, and in-hospital death. Procedural success was defined as the absence of procedural mortality and presence of correct positioning of a single THV into the proper anatomical location without interfering with a previously implanted mitral prosthesis. THV shift during valve deployment (upwards or downwards) was retrospectively assessed using recorded fluoroscopy. For SAPIEN XT and Evolut R, THV shift was defined as the movement of the ventricular THV edge. For the SAPIEN 3 valve, it was defined as the movement of the central balloon marker because SAPIEN 3 valves naturally become shortened on the ventricular side. THV shift was considered significant when the moving distance was at least 3 mm in fluoroscopy measurement referring to radiopaque markers of THV delivering devices. This was assessed by the agreement of two expert physicians blinded to the clinical information before the analysis. Predictors of THV shift were also assessed, defined per the Valve Academic Research Consortium 2 criteria [20].

We also compared pre- and postprocedural mitral prosthesis function and morphology. We compared pre- and postprocedural TTE parameters, such as mitral mean pressure gradient and regurgitation grade. We also evaluated stroke volume index and systolic pulmonary artery pressure. To assess the influence of TAVI on mitral prosthesis morphology, we compared the pre- and postprocedural housing area, maximal diameter, and minimal diameter using serial MDCT measurements.

The positional relationship between the newly implanted THV and mitral prosthesis was assessed. The postprocedural distance between THV and the mitral prosthesis housing (THV-mitral distance) was evaluated to assess the THV implantation depth by subtracting it from the preprocedural distance between the native aortic annulus and mitral prosthesis. The postprocedural angle between THV and the mitral prosthesis was measured via MDCT and compared with the preprocedural angle between LVOT and the mitral prosthesis, to assess the THV implantation angle in relation to the mitral prosthesis.

### Statistical analysis

Continuous variables are expressed as medians (interquartile range [IQR]). Paired continuous variables, such as pre- and postprocedural data, were compared using Wilcoxon’s signed-rank test. Categorical variables are expressed as numbers and percentages and were compared using the chi-square or Fisher’s exact test. Predictors of THV shift were assessed using logistic regression analysis. All statistical analyses were performed using SPSS version 24.0 (IBM Corp., Armonk, NY, USA). A *p*-value <0.05 indicated statistical significance.

## Results

### Patient and procedural characteristics

Of 3043 patients who underwent TAVI, 31 (1.0%) had previously undergone MVR prior to TAVI and were analyzed. Table 1 shows all baseline characteristics. Most were female (93.5%) and severely symptomatic, with New York Heart Association class III or IV (71%). Nineteen (61.3%) patients had preexisting atrial fibrillation and 12 (38.7%) patients had previous pacemaker implantation. All patients received oral anticoagulant therapy in the periprocedural period owing to preexisting mitral prosthesis and/or atrial fibrillation. Pre- and postprocedural CT images were acquired in all and 20 patients, respectively.

**Table 1:** Baseline characteristics.

	Patients
	(*n* = 31)
Clinical characteristics	
Age, years	81 (76–82)
Female sex	29 (93.5)
Body weight, kg	46.5 (40.0–52.3)
Height, cm	149.5 (144.0–154.0)
Body surface area, m^2^	1.40 (1.26–1.50)
NYHA class III or IV	22 (71.0)
STS PROM, %	8.20 (4.79–12.55)
Hypertension	18 (58.1)
Diabetes mellitus	9 (29.0)
Chronic kidney disease (stages 3–5)	22 (71.0)
Chronic obstructive pulmonary disease	7 (22.6)
Prior stroke or TIA	6 (19.4)
Peripheral artery disease	0 (0.0)
Coronary artery disease	9 (29.0)
Prior MI	2 (6.5)
Prior PMI	12 (38.7)
Atrial fibrillation	19 (61.3)
eGFR, mL/min/m^2^	42.7 (29.7–59.0)
BNP, ng/mL	208.7 (135.3–450.6)
Details of previous MVR	
Bioprosthetic valve	4 (12.9)
Years from surgery	14 (9–26)
Housing area, mm^2^	450.1 (437.7–514.9)
Housing maximal diameter, mm	24.3 (23.9–25.9)
Housing minimal diameter, mm	23.7 (23.3–25.4)
Distance between aortic annulus and housing of mitral prosthesis, mm	4.1 (3.2–5.7)
Angle between mitral prosthesis and LVOT, °	57 (52–62)
Mitral prosthesis housing protruding to LVOT	20 (64.5)
Echocardiographic findings	
AV maximal velocity, m/sec	4.0 (3.6–4.6)
AV mean pressure gradient, mmHg	39.0 (30.0–53.0)
Indexed AVA, cm^2^/m^2^	0.46 (0.36–0.60)
Ejection fraction, %	62.6 (53.0–67.4)
Stroke volume index, mL/m^2^	41.9 (34.6–56.6)
Systolic pulmonary artery pressure, mmHg	35.0 (29.5–47.8)
Mitral mean pressure gradient, mmHg	3.7 (2.3–4.1)
MR grade>2	1 (3.2)
AR grade>2	7 (22.6)
MDCT measurement of aortic valve complex	
Annulus area, mm^2^	366.6 (325.0–412.0)
Annulus perimeter, mm	69.4 (66.0–73.1)
Maximal annulus diameter, mm	24.5 (23.3–26.0)
Minimal annulus diameter, mm	19.2 (18.0–20.3)
LVOT area^a^, mm^2^	370.5 (334.3–428.4)
Procedural characteristics	
Type of THV	
SAPIEN XT, mm	
20	0 (0.0)
23	17 (54.8)
26	6 (19.4)
29	0 (0.0)
SAPIEN 3, mm	
20	1 (3.2)
23	4 (12.9)
26	0 (0.0)
29	0 (0.0)
Evolut R, mm	
23	0 (0.0)
26	2 (6.5)
29	1 (3.2)
%area oversizing^b^	19.9 (5.1–31.8)
Approach site	
Transfemoral	25 (80.6)
Transapical	6 (19.4)
Type of anesthesia	
General	25 (80.6)
Conscious sedation	6 (19.4)

Values are presented as median (interquartile range) or number (percentage).

NYHA = New York Heart Association; STS PROM = Society of Thoracic Surgeons Predictive Risk Of Mortality; TIA = transient ischemic attack; MI = myocardial infarction; PMI = pacemaker implantation; eGFR = estimated glomerular filtration rate; BNP = brain natriuretic peptide; MVR = mitral valve replacement; LVOT = left ventricular outflow tract; AV = aortic valve; AVA = aortic valve area; MR = mitral regurgitation; AR = aortic regurgitation; MDCT = multidetector computed tomography; THV = transcatheter heart valve.

^a^LVOT area was measured at 4 mm below the annulus plane.

^b^% area oversizing was calculated by nominal area of THV/native aortic annular area × 100 (%). The nominal area for SAPIEN 3 was defined as 328 mm^2^ for 20-mm THV and 409 mm^2^ for 23-mm THV according to the manufacturer. The nominal area for SAPIEN XT and Evolut R was defined as 415 mm^2^ for 23-mm THV, 531 mm^2^ for 26-mm THV, and 661 mm^2^ for 29-mm THV.

Overall, 27 (87.1%) and 4 (12.9%) patients had mechanical prosthesis and bioprosthesis, respectively. The median distance between the aortic annulus and mitral prosthesis housing was 4.1 (IQR, 3.2–5.7) mm. Protrusion of the mitral prosthesis housing into the LVOT was observed in 20 (64.5%) patients.

Overall, 23 (74.2%), 5 (16.1%), and 3 (9.7%) patients received SAPIEN XT, SAPIEN 3, and Evolut R valves for TAVI, respectively. Most TAVI procedures were performed via the transfemoral approach (*n* = 25, 80.6%) under general anesthesia (*n* = 25, 80.6%).

The clinical and procedural characteristics of individual patients are provided in S1 Table.

### Procedural outcomes and in-hospital complications

**Fig 3:**
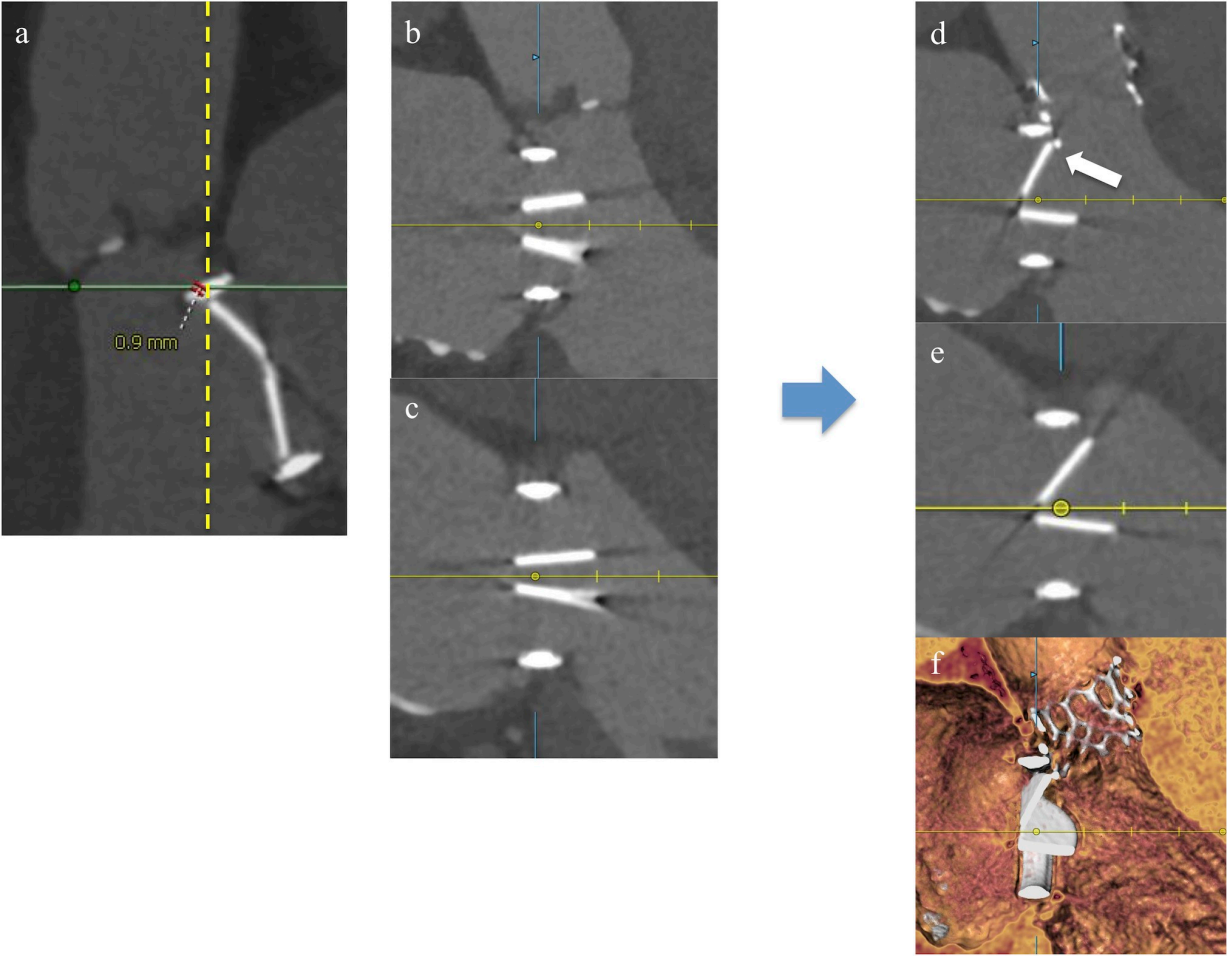
MDCT assessment of case #16. (a) Sagittal view of the aortic root (systolic-phase preprocedural MDCT). (b) Sagittal view of the mitral prosthesis (diastolic-phase preprocedural MDCT). (c) Perpendicular view of the mitral prosthesis leaflets (diastolic-phase preprocedural MDCT). (d) Sagittal view of the mitral prosthesis (diastolic-phase postprocedural MDCT). (e) Perpendicular view of the mitral prosthesis leaflets (diastolic-phase postprocedural MDCT). (f) Sagittal view of the mitral prosthesis (diastolic-phase postprocedural MDCT in the volume-rendering image).

Table 2 shows the procedural outcomes, including THV function assessed by postprocedural TTE, and in-hospital complications. Procedural success was achieved in all but one patient who developed THV interference with the mitral prosthesis. This patient had a 29-mm ATS bileaflet prosthesis (ATS Medical Inc., Minneapolis, MN, USA) implanted 9 years before TAVI (case #16 in S1 Table). After a 23-mm SAPIEN 3 was implanted transfemorally, the ventricular edge of the SAPIEN 3 partially occluded the opening of one leaflet of the ATS. The pre- and postprocedural MDCT findings are shown in Fig 3. The preprocedural MDCT revealed that the mitral prosthesis housing was close (0.9 mm) to the native aortic annulus and was protruding into the LVOT (Fig 3A). While the preprocedural MDCT demonstrated fully open mitral prosthesis leaflets (Fig 3B and 3C), postprocedurally the edge of the anterior leaflet impinged on the ventricular edge of the SAPIEN 3 valve and could not open completely (arrow in Fig 3D). Although the mitral mean pressure gradient increased from 1.0 to 5.0 mmHg and the grade of mitral regurgitation increased from 0 to 2 (case #16 in S1 Table), the patient was managed conservatively without exacerbation of heart failure and was discharged home. The patient was followed up for more than 1 year without any major adverse event. One of 3 patients who received Evolut R THV required recapturing and repositioning during deployment for implantation depth adjustment, not because of interference with the mitral prosthesis.

**Table 2:** Procedural outcomes and in-hospital complications.

	Patients
	(*n* = 31)
Procedural outcomes	
Procedural success	30 (96.8)
THV interference with mitral prosthesis	1 (3.2)
THV embolization	0 (0.0)
THV-in-THV deployment	0 (0.0)
THV shift during deployment	9 (29.0)
Upwards	8 (25.8)
THV function assessed by TTE	
Indexed EOA, cm^2^/m^2^	1.14 (0.88–1.35)
Severe patient-prosthesis mismatch	0 (0.0)
THV peak velocity, m/s	2.2 (1.9–2.4)
THV mean pressure gradient, mmHg	10.0 (7.4–12.0)
THV stenosis	2 (6.5)
PVL grade 3 or 4	1 (3.2)
Complications	
Disabled stroke	0 (0.0)
Bleeding	11 (35.5)
Life-threatening or disabling	0 (0.0)
Major	8 (25.8)
Minor	3 (9.7)
New or worsened cardiac conduction disturbance	2 (6.5)
New PMI	0 (0.0)
Acute kidney injury	2 (6.5)
Major vascular complication	3 (9.7)
Access site related	3 (9.7)
Aortic root injury	0 (0.0)
In-hospital death	1 (3.2)
Cardiovascular cause	0 (0.0)

Values are presented as median (interquartile range) or number (percentage).

THV = transcatheter heart valve; TTE = transthoracic echocardiography; EOA = effective orifice area; PVL = paravalvular leakage; PMI = pacemaker implantation.

Although THV embolization or THV-in-THV deployment was not noted, THV shift during deployment was observed in 9 patients (29.0%; 8 for upward and 1 for downward direction). All were implanted with balloon-expandable SAPIEN XT/3 and no THV shift was observed for the Evolut R; no significant relationship between THV shift occurrence and THV type was observed (*p* = 0.89).

Anatomically, a larger aortic area (odds ratio = 1.24 per 10 mm^2^, 1.03–1.49, *p* = 0.02) was found to be a predictor of THV shift. Implantation of a large-sized THV (26 or 29-mm for balloon-expandable and 29-mm for Evolut R) tended to increase THV shift (odds ratio = 6.80, 0.95–48.7, *p* = 0.06) (Table 3). This was consistent when cases were limited to balloon-expandable THVs (S2 Table). However, neither LVOT area, transfemoral approach, nor anatomical factors related to mitral prosthesis could predict THV shift. THV oversizing rate tended to be lower for patients with THV shift, but the difference was not statistically significant. The results regarding the THV shift predictor were consistent when cases were limited to upward THV shift. THV dysfunction, consisting of severe patient-prosthesis mismatch, THV stenosis, and paravalvular leakage grade 3 or 4, was unrelated to shift (0.0% for patients with THV shift vs. 13.6% for patients without THV shift, *p* = 0.54). The one reported in-hospital death (septic shock) was not procedure-related.

**Table 3:** Predictors of THV shift.

	Overall	THV shift (+)	THV shift (−)	*p*-value	OR	95% CI	*p*-value
	(*n* = 31)	(*n* = 9)	(*n* = 22)				
Bioprosthetic mitral valve	4 (12.9)	0 (0.0)	4 (18.2)	0.30			
Mitral prosthesis housing area, mm^2^	450.1 (437.7–514.9)	453.6 (434.1–521.1)	447.7 (439.4–515.3)	0.98	1.00	0.99–1.01	0.90
Mitral prosthesis housing protruding to LVOT	18 (58.1)	7 (77.8)	11 (50.0)	0.24	3.50	0.59–20.75	0.17
Distance between aortic annulus and housing of mitral prosthesis, mm	4.1 (3.2–5.7)	4.2 (3.5–5.0)	4.1 (2.8–6.0)	0.95	0.90	0.58–1.39	0.63
Angle between mitral prosthesis and LVOT, °	57 (52–62)	53 (47–65)	59 (54–61)	0.31	0.91	0.81–1.04	0.16
Aortic annulus area, mm^2^	366.6 (325.0–412.0)	408.0 (392.3–456.5)	348.5 (320.3–397.0)	0.02	1.24^a^	1.03–1.49	0.02
Aortic annulus ellipticity	1.33 (1.19–1.38)	1.25 (1.19–1.35)	1.35 (1.19–1.40)	0.21	0.01	0.00–22.44	0.24
LVOT area, mm^2^	370.5 (334.3–428.4)	388.8 (339.0–464.4)	359.3 (333.6–408.8)	0.41	1.02^a^	0.94–1.12	0.62
Large-sized THV^b^	7 (22.6)	4 (44.4)	3 (13.6)	0.15	6.80	0.95–48.7	0.06
%area oversizing, %^c^	19.9 (5.1–31.8)	5.1 (1.8–29.1)	26.1 (10.6–32.1)	0.11	0.007	0.00–2.34	0.10
Transfemoral approach	25 (80.6)	6 (66.7)	19 (86.4)	0.32	0.32	0.05–2.00	0.22
Balloon-expandable THV	28 (90.3)	9 (100.0)	19 (86.4)	0.89			

Values are presented as median (interquartile range) or number (percentage).

THV = transcatheter heart valve; LVOT = left ventricular outflow tract; OR = odds ratio; CI = confidence interval.

^a^OR for aortic annulus area and LVOT area are per 10 mm^2^.

^b^Large-sized THV corresponds to 26 or 29-mm THV for balloon-expandable THVs and 29-mm THV for Evolut R.

^c^%area oversizing was calculated by nominal area of THV/native aortic annular area ×100 (%).The nominal area for SAPIEN 3 was defined as 328 mm^2^ for 20-mm THV and 409 mm^2^ for 23-mm THV, according to the manufacturer. The nominal area for SAPIEN XT and Evolut R was defined as 415 mm^2^ for 23-mm THV, 531 mm^2^ for 26-mm THV, and 661 mm^2^ for 29-mm THV.

### Pre- and postprocedural mitral prosthesis function and morphology

Table 4A shows pre- and postprocedural TTE-assessed mitral prosthesis function. The mean pressure gradient significantly increased from 3.7 to 4.3 mmHg (*p* = 0.002); mitral regurgitation grade did not significantly change. Increased mitral mean pressure gradient and unchanged regurgitation grade were consistent if case #16 with THV interference was excluded (S3 Table). Follow-up TTE data obtained 6 months after the procedure were available for 20 cases (S4 Table). At this time point, in line with other parameters, the change in mitral pressure gradient was not statistically significant when pre- and postprocedural TTE were compared.

**Table 4:** Pre- and postprocedural mitral prosthesis function and morphology.

**A: Functional assessment of mitral prosthesis by TTE**			
	Preprocedure	Postprocedure	*p*-value
	(*n* = 31)	(*n* = 31)	
Mean pressure gradient, mmHg	3.7 (2.3–4.1)	4.3 (3.0–5.0)	0.002
Stroke volume index, mL/m^2^	41.9 (34.6–56.6)	50.5 (33.5–58.2)	0.72
Systolic pulmonary artery pressure, mmHg	35.0 (29.5–47.8)	39.5 (32.3–58.3)	0.13
MR grade			
0	8 (25.8)	7 (22.6)	0.89
1	13 (41.9)	16 (51.6)	
2	9 (29.0)	7 (22.6)	
3	1 (3.2)	1 (3.2)	
4	0 (0.0)	0 (0.0)	
**B: Morphologic assessment of mitral prosthesis by MDCT measurement**			
	Preprocedure	Postprocedure	*p*-value
	(*n* = 20)	(*n* = 20)	
Housing area, mm^2^	448.0 (434.2–516.0)	450.6 (429.7–517.8)	0.68
Housing maximal diameter, mm	24.5 (23.8–25.9)	24.6 (23.9–26.1)	0.24
Housing minimal diameter, mm	23.7 (23.1–25.4)	23.6 (22.8–25.3)	0.09

Values are presented as median (interquartile range) or number (percentage).

TTE = transthoracic echocardiography; MR = mitral regurgitation; MDCT = multidetector computed tomography.

Pre- and postprocedural MDCT-evaluated mitral prosthesis morphology did not change, but it trended toward decreased housing minimal diameter postprocedurally (Table 4B).

### Positional relationship between newly implanted THV and mitral prosthesis

Pre- and postprocedural MDCT measurements for the 20 patients with serial MDCT examinations showed a significantly larger angle between THV or the native aortic valve and the mitral prosthesis housing postprocedurally (median 64° [IQR, 54–65°] postprocedurally vs. 61° [IQR, 58–69°] preprocedurally; *p* = 0.03). The median leaning angle, calculated by subtracting the preprocedural from the postprocedural angle, was 4° (IQR, −1 to 8).

The median THV-mitral distance was 2.6 (IQR, 1.8–4.2) mm. The median THV implantation depth, calculated by the THV-mitral distance and the preprocedural distance between the aortic annulus and mitral prosthesis, was 1.6 (IQR, 0.3–2.5) mm.

For case #16 (with THV interference), the distance between the mitral prosthesis and THV was −3.4 mm (i.e., the two prostheses overlapped). For case #24, the THV implantation depth was −1.0 mm; however, the THV was adequately anchored within the annulus and was not embolized. For the other cases, the THV implantation depth was measured as a positive value and was within the preprocedural distance between the mitral prosthesis and native annulus (S1 Table).

## Discussion

We demonstrated that 1) TAVI for the patients with previously implanted mitral prosthesis, including the new-generation balloon- (SAPIEN 3) and self-expandable (Evolut R) devices, was safe, without any adverse impact on THV function and correct positioning; 2) THV shift was associated with a large native aortic annulus and, consequently, had a trend for large-sized THV; and 3) the mean transmitral pressure gradient was significantly increased after TAVI but no significant morphological change in previously implanted mitral prosthesis was confirmed by postprocedural MDCT analysis. To our knowledge, this is the first study to demonstrate, via postprocedural MDCT, the absence of a significant morphological change in the mitral prosthesis.

### Procedural outcomes and risk evaluation

This study had satisfactory procedural success (96.8%). Although one case of THV interference with the mitral prosthesis occurred, the patient had a benign course, unlike previous cases [7]. In this particular case of our study, the THV impinged on one of the two mitral mechanical leaflets, partially interfering with the opening of the leaflet. Consequently, the other leaflet worked normally and it had minimal impact on hemodynamics. Nevertheless, it is imperative to avoid this situation as it may become critical. Regarding the potential risk of interference retrospectively assessed by MDCT, the distance between the mitral prosthesis and native aortic annulus was very short (0.9 mm) and the edge of the mitral prosthesis housing significantly protruded into the LVOT. This anatomical feature might have led to interference of the two prostheses. Moreover, the unique behavior of SAPIEN 3 during deployment, characterized by the significant shortening of the ventricular edge, might have made implantation in a correct position more challenging. Consequently, careful preprocedural MDCT assessment is necessary to ascertain if the risk of interference of two prostheses is manageable. In this condition, a repositionable-type THV, such as Evolut R, might be helpful as the limited number of patients in our study achieved procedural success. Moreover, the type of previously implanted mitral prosthesis appears to be important because the degree of protrusion into the LVOT differed by the type of mitral prosthesis [4,5].

Although no THV embolization occurred, THV shift during deployment, which can predispose to embolization [21], occurred in 29.0% of the patients. The significant predictor of this phenomenon was a larger aortic annulus, possibly because it is treated with a large-sized THV. Larger THVs have a taller stent frame that may easily be affected by preexisting mitral prosthesis during deployment. Thus, operators should consider slow THV deployment in such situations. Other factors, such as a short distance between the aortic annulus and mitral prosthesis, mitral prosthesis protrusion into the LVOT, bioprosthetic mitral valve, THV oversizing, and transfemoral approach [4,10] were not identified as predictors of THV shift or embolization. Moreover, as described previously [6], the angle between the LVOT and mitral prosthesis did not influence THV shift. However, this result should be cautiously interpreted because we could not directly examine the risk of embolization, and THV shift could be affected by the operator (pulling or pushing the valve during deployment). The prevalence of THV shift that we observed is higher than the 7.5% prevalence reported from a previous study [14]. However, direct comparison is not possible because no standard definition of THV shift has been established, and so the definition may vary between studies. Moreover, although THV shift is considered to be one cause of THV embolization [21], it did not trigger THV embolization in either the present or previous study [14]. Further studies involving larger numbers of patients could provide insight into the clinical impact of the THV shift phenomenon.

With regard to in-hospital complications, a relatively high prevalence of major bleeding (25.8%) was observed in the present study, and this may be attributable to the fact that all patients in this study received oral anticoagulant therapy in the periprocedural period. Furthermore, the prevalences of female sex and atrial fibrillation were high in our cohort, and these are known risk factors for bleeding complications [22–24].

### Pre- and postprocedural function and morphology

Postprocedural mitral prostheses morphology did not significantly change after TAVI, probably owing to the rigidity of the housing. Thus, there was also no change in mitral regurgitation grade. Postprocedural mitral mean pressure gradient was significantly higher. However, considering the increasing trend of stroke volume and unchanged mitral prosthesis morphology, this change could be due to decreased left ventricular diastolic pressure and increased blood flow through the mitral prosthesis, reflecting hemodynamic improvement after TAVI rather than impaired mitral prosthesis performance [25,26].

### Positional relationship between newly implanted THV and mitral prosthesis

The THV implantation depth was less than the distance between the mitral prosthesis and native annulus, except in case #16, which means the THV was properly implanted without interference with the mitral prosthesis. The operators successfully managed the position of THV that was very close to the mitral prosthesis housing (median 2.6 mm), by applying a technique described in previous reports, such as slow THV deployment [4,5,9,11]. Moreover, the predominant use of general anesthesia in the present study (80.6%) might reflect the operators’ consideration that general anesthesia with controlled ventilation and transesophageal echocardiographic guidance can make THV deployment more secure in this challenging situation [11]. The preprocedural distance between the aortic annulus and mitral prosthesis in the present study was shorter than that in a previous report [6] possibly because of the different measurement technique; our measurement was from the virtual basal ring of the aortic annulus and was more ventricular because we always aim for this level for THV deployment.

A larger postprocedural angle between the THV and mitral prosthesis compared with the preprocedural angle between the LVOT and mitral prosthesis indicates that implanted THVs lean away from the mitral prostheses. Although the present study did not clarify whether THV leaning was due to the presence of mitral prosthesis or to the stiff wire in the left ventricle, a deeper implantation depth at the right coronary cusp side would aid the prevention of THV embolization. As in case #24, the THV implantation depth was -1.0 mm but the THV was not embolized, possibly because the THV leaned by 8° and, consequently, THV could be properly anchored at the right coronary cusp side.

### Limitations

The present study has several limitations. First, it was an observational study; thus, unexpected factors such as operators’ experience and preference might have influenced the results.

Second, this study included a limited number of patients with a mitral prosthesis. Although the OCEAN-TAVI registry is one of the largest registries in Japan and the present study included a relatively large number of patients and new-generation THVs compared with previous related reports, TAVI in the presence of a mitral prosthesis is a rare situation. More patients would be necessary for a more precise analysis of MDCT measurement or specific procedural risks.

Finally, with regards to the MDCT analysis, we could not evaluate the potential association between THV shift and the calcification amount of the aortic valve complex because we could not quantify the calcification precisely due to the artifact arising from the mitral prosthesis.

## Conclusions

TAVI in the presence of mitral prosthesis was generally feasible. A larger aortic annulus predicted THV shift during deployment. Serial MDCT demonstrated favorable THV positioning and unchanged mitral prosthesis morphology after TAVI.

## Supporting information

S1 Fig Pre- and postprocedural MDCT measurement of bioprosthetic mitral valves. (a) Size of the mitral prosthesis housing. (b) The distance between the aortic annulus and mitral prosthesis housing. (c) The distance between the newly implanted THV and mitral prosthesis housing. (d) The angle between the mitral prosthesis and LVOT. (e) The angle between the mitral prosthesis and newly implanted THV. (f) Assessment of mitral prosthesis protrusion to the LVOT. LVOT = left ventricular outflow tract; MDCT = multidetector computed tomography; THV = transcatheter heart valve.
(TIFF)

S1 Table Clinical and procedural characteristics of individual patients. Y = yes; N = no; NA = not available; MVR = mitral valve replacement; TAP = tricuspid annuloplasty; LAA = left atrial appendage; PVI = pulmonary venous isolation; CABG = coronary artery bypass graft surgery; OAC = open aortic commissurotomy; AVP = aortic valve plasty; CEP = Carpentier-Edwards Perimount; THV = transcatheter heart valve; TF = transfemoral; TA = transapical; TTE = transthoracic echocardiography; PG = pressure gradient; MR = mitral regurgitation; PVL = paravalvular leakage; MDCT = multidetector computed tomography; LVOT = left ventricular outflow tract.
(PDF)

S2 Table Predictors of THV shift (balloon-expandable THVs). Values are presented as median (interquartile range) or number (percentage). THV = transcatheter heart valve; LVOT = left ventricular outflow tract; OR = odds ratio; CI = confidence interval. ^a^OR for aortic annulus area and LVOT area are per 10 mm^2^.
^b^Large-sized THV corresponds to 26- or 29-mm THV for balloon-expandable THVs and 29-mm THV for Evolut R.
^c^%area oversizing was calculated by nominal area of THV/native aortic annular area × 100 (%). The nominal area for SAPIEN 3 was defined as 328 mm^2^ for 20-mm THV and 409 mm^2^ for 23-mm THV according to the manufacturer. The nominal area for SAPIEN XT was defined as 415 mm^2^ for 23-mm THV and 531 mm^2^ for 26-mm THV.
(PDF)

S3 Table Functional assessment of mitral prosthesis by TTE (case #16 excluded). Values are presented as median (interquartile range) or number (percentage). MR = mitral regurgitation.
(PDF)

S4 Table Follow-up TTE data for mitral prosthesis function. Values are presented as median (interquartile range) or number (percentage). MR = mitral regurgitation.
^a^*p*-value for preprocedure vs. postprocedure.
^b^*p*-value for preprocedure vs. 6 months after the procedure.
^c^*p*-value for postprocedure vs. 6 months after the procedure.
(PDF)
