# Supplementary figures and images for: Previously implanted mitral surgical prosthesis in patients undergoing transcatheter aortic valve implantation: Procedural outcome and morphologic assessment using multidetector computed tomography
Source: PLoS One. 2019 Dec 26;14(12):e0226512. doi: 10.1371/journal.pone.0226512 (PMC6932792; doi:10.1371/journal.pone.0226512)

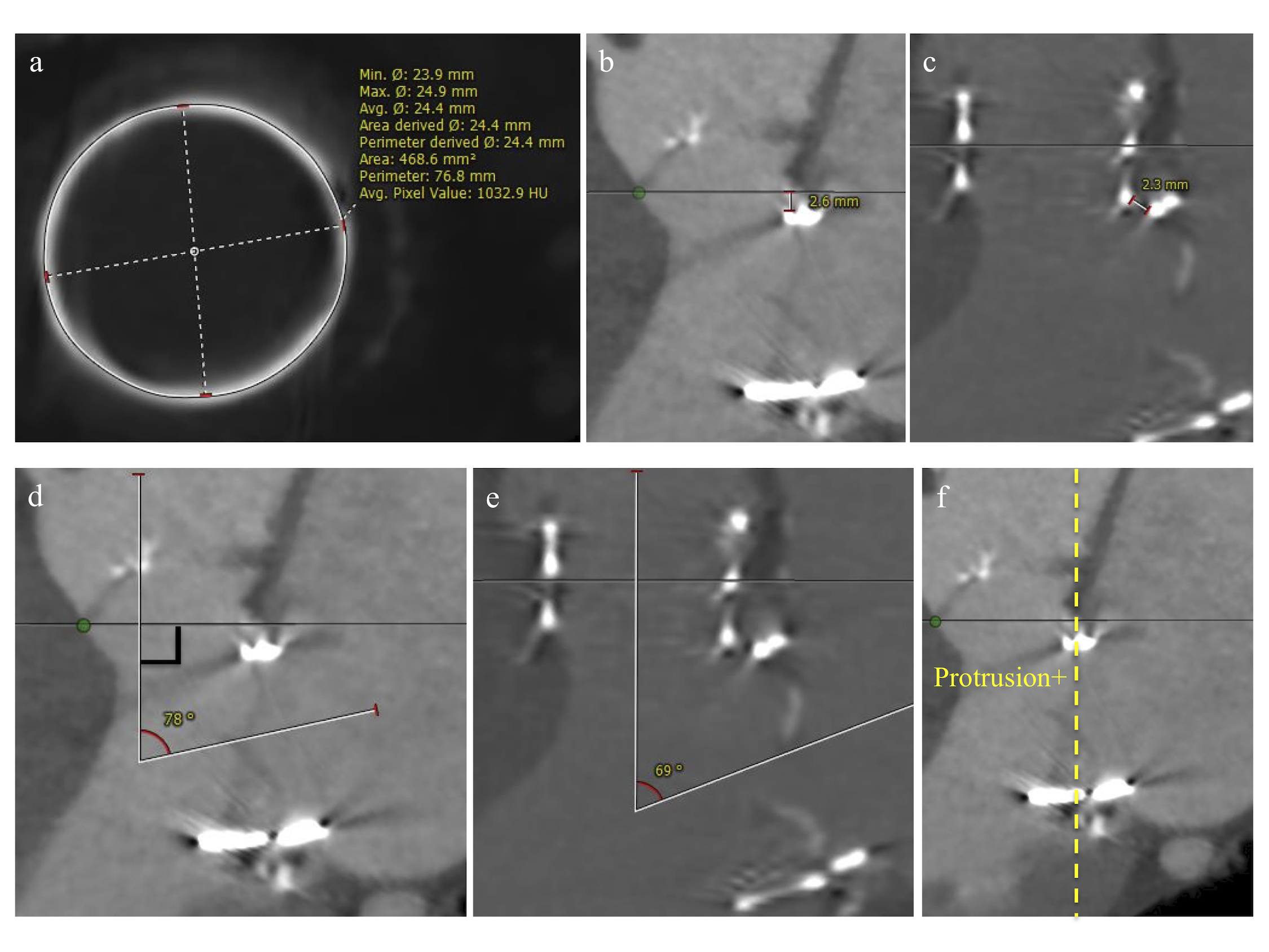

Supplement: S1 Fig — (a) Size of the mitral prosthesis housing. (b) The distance between the aortic annulus and mitral prosthesis housing. (c) The distance between the newly implanted THV and mitral prosthesis housing. (d) The angle between the mitral prosthesis and LVOT. (e) The angle between the mitral prosthesis and newly implanted THV. (f) Assessment of mitral prosthesis protrusion to the LVOT. LVOT = left ventricular outflow tract; MDCT = multidetector computed tomography; THV = transcatheter heart valve. (TIFF) [file pone.0226512.s001.tiff]
