# Supplementary material for: Previously implanted mitral surgical prosthesis in patients undergoing transcatheter aortic valve implantation: Procedural outcome and morphologic assessment using multidetector computed tomography
Source: PLoS One. 2019 Dec 26;14(12):e0226512. doi: 10.1371/journal.pone.0226512 (PMC6932792; doi:10.1371/journal.pone.0226512)
Supplement: S1 Table — Y = yes; N = no; NA = not available; MVR = mitral valve replacement; TAP = tricuspid annuloplasty; LAA = left atrial appendage; PVI = pulmonary venous isolation; CABG = coronary artery bypass graft surgery; OAC = open aortic commissurotomy; AVP = aortic valve plasty; CEP = Carpentier-Edwards Perimount; THV = transcatheter heart valve; TF = transfemoral; TA = transapical; TTE = transthoracic echocardiography; PG = pressure gradient; MR = mitral regurgitation; PVL = paravalvular leakage; MDCT = multidetector computed tomography; LVOT = left ventricular outflow tract. (PDF) [file pone.0226512.s002.pdf]

S1 Table. Clinical and procedural characteristics of individual patients

									Preprocedural TTE		Postprocedural TTE				Distance from mitral prosthesis, mm								
Case #	Sex	Age, years	Previous surgery	Years from MVR	Mitral prosthesis		Implanted THV	Approach site	Mitral mean PG, mmHg	MR grade	Mitral mean PG, mmHg	MR grade	THV dysfunction	Postprocedural MDCT available	From housing to aortic annulus	From stem to aortic annulus	From housing to implanted THV	THV implantation depth, mm	Angle between mitral prosthesis and LVOT,°	Angle between mitral prosthesis and THV,°	Housing protruding to LVOT	THV shift	Procedure success
1	Female	82	MVR + TAP + LAA ligation	11	Mechanical	St. Jude Medical 27 mm	SAPIEN XT 23 mm	TF	4.1	1	4.5	1	N	Y	4.2		1.7	2.5	66	73	Y	Upward	Y
2	Female	82	MVR + TAP + Maze	12	Mechanical	St. Jude Medical 27 mm	SAPIEN XT 26 mm	TF	3.4	2	2.7	2	N	Y	5.4		5.2	0.2	52	62	N	N	Y
3	Female	77	MVR + TAP	3	Bioprosthetic	CEP plus 25 mm	SAPIEN XT 23 mm	TF	5.0	1	5.3	1	THV stenosis	Y	3.3	9.6	2.4	0.9	65	58	Y	N	Y
4	Female	80	MVR + TAP	14	Mechanical	St. Jude Medical 27 mm	SAPIEN XT 26 mm	TF	3.0	2	3.7	1	N	Y	3.7		2.1	1.6	64	67	Y	Downward	Y
5	Female	81	MVR + TAP + PVI	5	Bioprosthetic	CEP 25 mm	SAPIEN XT 23 mm	TF	2.5	1	5.9	2	N	Y	2.6	7.6	2.6	0.0	78	81	Y	N	Y
6	Female	81	MVR	26	Mechanical	Bjork-Shiley (size unknown)	SAPIEN XT 23 mm	TA	2.3	1	2.2	1	N	Y	6.7		5.1	1.6	60	66	Y	N	Y
7	Female	72	MVR	9	Mechanical	St. Jude Medical 25 mm	SAPIEN XT 23 mm	TA	5.1	2	6.6	2	N	Y	3.7		2.6	1.1	62	59	Y	N	Y
8	Male	88	MVR	26	Mechanical	Unknown (bileaflet)	SAPIEN XT 26 mm	TF	2.1	1	2.8	1	N	Y	3.2		3.1	0.1	65	64	Y	Upward	Y
9	Female	89	MVR + TAP	36	Mechanical	Omniscience 29 mm	SAPIEN3 20 mm	TF	4.0	1	4.3	1	THV stenosis	N	5.0		NA	NA	61	NA	Y	N	Y
10	Female	81	MVR	30	Mechanical	Duromedics 27 mm	SAPIEN3 23 mm	TF	2.0	2	1.7	2	N	Y	4.1		2.5	1.6	63	63	Y	N	Y
11	Female	86	MVR	10	Mechanical	Unknown (bileaflet)	SAPIEN XT 23 mm	TF	5.9	2	5.0	1	N	Y	6.6		4.3	2.3	55	67	Y	N	Y
12	Female	64	MVR	31	Mechanical	Bjork-Shiley 29 mm	SAPIEN3 23 mm	TF	4.0	2	5.0	2	N	N	5.4		NA	NA	48	NA	Y	Upward	Y
13	Female	77	MVR	20	Mechanical	Unknown (bileaflet)	SAPIEN XT 26 mm	TF	2.0	2	4.0	0	N	Y	5.7		0.4	5.3	52	48	N	N	Y
14	Female	79	MVR + TAP	25	Mechanical	Carbomedics 27 mm	SAPIEN XT 23 mm	TF	4.0	0	5.0	0	N	Y	9.2		4.2	5.0	57	58	N	N	Y
15	Female	76	MVR + CABG	4	Bioprosthetic	Mosaic 25 mm	SAPIEN XT 23 mm	TF	3.0	0	3.0	0	N	Y	2.4	9.9	1.0	1.4	68	77	N	N	Y
16	Female	75	MVR	9	Mechanical	ATS 29 mm	SAPIEN3 23 mm	TF	1.0	0	5.0	2	N	Y	0.9		-3.4	4.3	61	66	Y	N	N
17	Female	82	MVR	30	Mechanical	Bjork-Shiley 27 mm	SAPIEN XT 23 mm	TF	4.0	1	4.0	1	N	Y	4.5		4.0	0.5	53	43	Y	Upward	Y
18	Female	77	MVR	15	Mechanical	Carbomedics 25 mm	Evolut R 26 mm	TF	8.0	1	7.0	1	N	N	5.5		NA	NA	57	NA	Y	N	Y
19	Female	69	MVR + Maze	18	Mechanical	Carbomedics 27 mm	Evolut R 26 mm	TF	4.0	1	5.0	1	N	Y	9.2		4.5	4.7	50	56	N	N	Y
20	Female	81	MVR + CABG	13	Mechanical	St. Jude Medical 27 mm	SAPIEN XT 23 mm	TA	3.0	1	7.0	0	N	N	2.9		NA	NA	50	NA	N	N	Y
21	Female	89	MVR + OAC	12	Mechanical	St. Jude Medical 27 mm	SAPIEN XT 23 mm	TF	2.8	0	2.8	0	N	N	5.8		NA	NA	45	NA	N	Upward	Y
22	Female	74	MVR	20	Mechanical	Unknown (bileaflet)	SAPIEN XT 23 mm	TF	2.1	0	3.2	0	N	N	6.6		NA	NA	61	NA	Y	N	Y
23	Female	76	MVR + LAA ligation + Maze	14	Mechanical	St. Jude Medical 29 mm	SAPIEN XT 23 mm	TF	1.6	0	2.5	0	Moderate PVL	N	3.4		NA	NA	52	NA	Y	N	Y
24	Male	82	MVR	32	Mechanical	Medtronic Hall 27 mm	SAPIEN XT 26 mm	TA	5.0	1	5.0	1	N	Y	3.2		4.2	-1.0	42	50	Y	Upward	Y
25	Female	86	MVR + TAP	22	Mechanical	St. Jude Medical 29 mm	Evolut R 29 mm	TF	4.0	2	4.0	2	N	Y	5.8		4.1	1.7	55	58	N	N	Y
26	Female	72	MVR	33	Mechanical	Bjork-Shiley 27 mm	SAPIEN XT 23 mm	TF	5.2	1	5.6	1	N	N	3.6		NA	NA	57	NA	N	N	Y
27	Female	86	MVR + Maze	12	Bioprosthetic	CEP 27 mm	SAPIEN XT 23 mm	TF	7.2	3	11.2	3	N	Y	2.3	10.3	2.3	0.0	61	69	Y	N	Y
28	Female	89	MVR + AVP	5	Mechanical	St. Jude Medical 25 mm	SAPIEN XT 23 mm	TF	3.8	0	5.0	1	N	N	4.1		NA	NA	60	NA	Y	N	Y
29	Female	76	MVR + TAP	7	Mechanical	St. Jude Medical 27 mm	SAPIEN XT 23 mm	TA	1.8	2	1.9	1	N	N	3.8		NA	NA	56	NA	N	Upward	Y
30	Female	81	MVR	21	Mechanical	St. Jude Medical 29 mm	SAPIEN XT 26 mm	TA	2.8	0	3.4	1	N	N	4.5		NA	NA	53	NA	Y	Upward	Y
31	Female	82	MVR + LAA ligation	9	Mechanical	St. Jude Medical 27 mm	SAPIEN3 23 mm	TF	3.7	1	3.2	1	N	Y	2.5		1.0	1.5	58	78	N	N	Y

Y=yes; N=no; NA=not available; MVR=mitral valve replacement; TAP=tricuspid annuloplasty; LAA=left atrial appendage; PVI=pulmonary venous isolation; CABG=coronary artery bypass graft surgery; OAC=open aortic commissurotomy; AVP=aortic valve plasty; CEP=Carpenter-Edwards Perimount; THV=transcatheter heart valve; TF=transfemoral; TA=transapical; TTE=transthoracic echocardiography; PG=pressure gradient; MR=mitral regurgitation; PVL=paravalvular leakage; MDCT=multidetector computed tomography; LVOT=left ventricular outflow tract.
