# Supplementary material for: Previously implanted mitral surgical prosthesis in patients undergoing transcatheter aortic valve implantation: Procedural outcome and morphologic assessment using multidetector computed tomography
Source: PLoS One. 2019 Dec 26;14(12):e0226512. doi: 10.1371/journal.pone.0226512 (PMC6932792; doi:10.1371/journal.pone.0226512)
Supplement: S2 Table — Values are presented as median (interquartile range) or number (percentage). THV = transcatheter heart valve; LVOT = left ventricular outflow tract; OR = odds ratio; CI = confidence interval. aOR for aortic annulus area and LVOT area are per 10 mm2. bLarge-sized THV corresponds to 26- or 29-mm THV for balloon-expandable THVs and 29-mm THV for Evolut R. c%area oversizing was calculated by nominal area of THV/native aortic annular area × 100 (%). The nominal area for SAPIEN 3 was defined as 328 mm2 for 20-mm THV and 409 mm2 for 23-mm THV according to the manufacturer. The nominal area for SAPIEN XT was defined as 415 mm2 for 23-mm THV and 531 mm2 for 26-mm THV. (PDF) [file pone.0226512.s003.pdf]

S2 Table. Predictors of THV shift (Balloon-expandable THVs)

	Overall (*n*=28)	THV shift (+) (*n*=9)	THV shift (-) (*n*=19)	*p*-value	OR	95% CI	*p*-value
Bioprosthetic mitral valve	4 (14.3)	0(0.0)	4 (21.1)	0.27			
Mitral prosthesis housing area, mm^2	451.9 (437.0-514.7)	453.6 (434.1-521.1)	449.5 (437.7-514.9)	1.00	1.00	0.99-1.01	1.00
Mitral prosthesis housing protruding to LVOT	17(60.7)	7 (77.8)	10 (52.6)	0.25	3.15	0.52-19.27	0.21
Distance between aortic annulus and housing of mitral prosthesis, mm	4.0 (3.2-5.4)	4.2 (3.5-5.0)	3.7 (2.6-5.7)	0.61	1.00	0.63-1.59	0.63
Angle between mitral prosthesis and LVOT, deg.	59 (52-63)	53 (47-65)	60 (55-62)	0.26	0.90	0.79-1.03	0.11
Aortic annulus area, mm^2	372.9 (321.5-412.0)	408.0 (392.3-456.5)	346.0 (320.1-392.0)	0.02	1.31[a]	1.05-1.64	0.02
Aortic annulus ellipticity	1.33 (1.19-1.39)	1.25 (1.19-1.35)	1.36 (1.19-1.42)	0.12	0.004	0.00-10.89	0.17
LVOT area, mm^2	372.0 (332.5-426.8)	388.8 (339.0-464.4)	367.3 (331.6-399.7)	0.36	1.05[a]	0.94-1.17	0.37
Large-sized THV[b]	6 (21.4)	4 (44.4)	2 (10.5)	0.06	5.07	0.84-30.41	0.08
Transfemoral approach	25 (80.6)	6 (66.7)	16 (84.2)	0.35	0.38	0.06-2.40	0.30
% area oversizing[c]	19.4 (5.0-29.4)	5.1 (1.8-29.1)	19.9 (7.9-29.6)	0.25	0.02	0.00-9.15	0.21

Values are presented as median (interquartile range) or number (percentage).
THV=transcatheter heart valve; LVOT=left ventricular outflow tract; OR=odds ratio; CI=confidence interval.
[a]OR for aortic annulus area and LVOT area are per 10 mm^2.
[b]Large-sized THV corresponds to 26 or 29-mm THV for balloon-expandable THVs and 29-mm THV for Evolut R.
[c]%area oversizing was calculated by nominal area of THV/native aortic annular area x100 (%). The nominal area for SAPIEN 3 was defined as 328 mm^2 for 20-mm THV and 409 mm^2 for 23-mm THV according to the manufacturer. The nominal area for SAPIEN XT was defined as 415 mm^2 for 23-mm THV and 531 mm^2 for 26-mm THV.
