# Supplementary material for: Previously implanted mitral surgical prosthesis in patients undergoing transcatheter aortic valve implantation: Procedural outcome and morphologic assessment using multidetector computed tomography
Source: PLoS One. 2019 Dec 26;14(12):e0226512. doi: 10.1371/journal.pone.0226512 (PMC6932792; doi:10.1371/journal.pone.0226512)
Supplement: S3 Table — Values are presented as median (interquartile range) or number (percentage). MR = mitral regurgitation. (PDF) [file pone.0226512.s004.pdf]

S3 Table. Functional assessment of mitral prosthesis by TTE (Case #16 excluded)

	Preprocedure (n=30)	Postprocedure (n=30)	p-value
Mean pressure gradient, mmHg	3.8 (2.5-4.3)	4.2 (3.0-5.1)	0.004
Stroke volume index, mL/m^2	42.1 (34.5-56.8)	51.6 (35.9-58.3)	0.61
Systolic pulmonary artery pressure, mmHg	35.0 (29.3-49.2)	39.0 (32.1-53.0)	0.17
MR grade			
0	7 (23.3)	7 (23.3)	0.82
1	13 (43.3)	16 (53.3)	
2	9 (30.0)	6 (20.0)	
3	1 (3.3)	1 (3.3)	
4	0 (0.0)	0 (0.0)	

Values are presented as median (interquartile range) or number (percentage).
MR=mitral regurgitation
