# Supplementary material for: Previously implanted mitral surgical prosthesis in patients undergoing transcatheter aortic valve implantation: Procedural outcome and morphologic assessment using multidetector computed tomography
Source: PLoS One. 2019 Dec 26;14(12):e0226512. doi: 10.1371/journal.pone.0226512 (PMC6932792; doi:10.1371/journal.pone.0226512)
Supplement: S4 Table — Values are presented as median (interquartile range) or number (percentage). MR = mitral regurgitation. ap-value for preprocedure vs. postprocedure. bp-value for preprocedure vs. 6 months after the procedure. cp-value for postprocedure vs. 6 months after the procedure. (PDF) [file pone.0226512.s005.pdf]

**S4 Table. Follow-up TTE data for mitral prosthesis function.**

| | Preprocedure (*n*=20) | Postprocedure (*n*=20) | 6 months after the procedure (*n*=20) | *p*-value[a] | *p*-value[b] | *p*-value[c] |
|---|---|---|---|---|---|---|
| Mean pressure gradient, mmHg | 3.9 (2.2-5.0) | 4.8 (3.3-5.0) | 3.3 (3.0-5.7) | 0.002 | 0.18 | 0.18 |
| Stroke volume index, ml/m2 | 40.2 (34.7-56.2) | 50.1 (34.3-55.8) | 43.6 (38.3-51.9) | 0.60 | 0.74 | 0.20 |
| Systolic pulmonary artery pressure, mmHg | 33.0 (29.0-50.6) | 42.0 (34.7-53.0) | 39.0 (33.3-44.7) | 0.09 | 0.93 | 0.19 |
| MR grade | | | | | | |
| 0 | 6 (30.0) | 4 (20.0) | 5 (25.0) | 0.44 | 0.59 | 0.98 |
| 1 | 6 (30.0) | 11 (55.0) | 10 (50.0) | | | |
| 2 | 7 (35.0) | 4 (20.0) | 4 (20.0) | | | |
| 3 | 1 (5.0) | 1 (5.0) | 1 (5.0) | | | |
| 4 | 0 (0.0) | 0 (0.0) | 0 (0.0) | | | |

Values are presented as median (interquartile range) or number (percentage).
MR=mitral regurgitation.
[a]*p*-value for preprocedure vs. postprocedure.
[b]*p*-value for preprocedure vs. 6 months after the procedure.
[c]*p*-value for postprocedure vs. 6 months after the procedure.
